# Editorial: Progress in clinical and mechanistic studies of severe respiratory viral infections in children

**DOI:** 10.3389/fped.2025.1703217

**Published:** 2025-10-01

**Authors:** Dingle Yu, Xiaohui Wang, Gen Lu

**Affiliations:** ^1^ Department of Respiration, Shenzhen Children’s Hospital, Shenzhen University, Shantou University Medical College, Shenzhen, Guangdong, China; ^2^ Guangzhou Institute of Pediatrics, Guangzhou Women and Children’s Medical Center, State Key Laboratory of Respiratory Disease, Guangzhou Medical University, Guangzhou, China; ^3^ Department of Respiratory Medicine, Guangzhou Women and Children’s Medical Center, Guangzhou Medical University, Guangzhou, Guangdong, China

**Keywords:** respiratory virus, infection, child, clinical feature, pathological mechanism

**Editorial on the Research Topic**
Progress in clinical and mechanistic studies of severe respiratory viral infections in children

Acute viral respiratory infections remain a leading cause of under-five mortality globally (1), yet targeted therapeutics are still lacking. In recent years, the emergence and recurrent outbreaks of respiratory pathogens such as novel coronaviruses (2), influenza viruses, respiratory syncytial virus (RSV) (3), and adenoviruses have posed a significant threat to pediatric populations. The immune system, alveoli, distal vasculature, and nervous system of newborns and preschool children remain underdeveloped, resulting in distinct immunological characteristics and regulatory mechanisms in their lungs compared to adults (4). While clinical manifestations in children are well-documented, critical knowledge gaps persist regarding pathogenesis, diagnostic biomarkers, therapeutic targets, immune mechanisms, and long-term sequelae. Exploring these scientific questions not only reveals the fundamental causes of disease variation in potential future viral pandemics but also provides new strategies for addressing urgent clinical challenges in the prevention and treatment of severe novel pneumonia. This ultimately enables precise interventions and accelerating the development of preventive and therapeutic vaccines.

This research topic focuses on severe viral respiratory infections in children, integrating both clinical observations and mechanistic investigations. It specifically targets the complexities of severe viral pneumonia and invites original research contributions exploring critical areas including microbe-host interactions, pathogenesis, diagnostic biomarkers, novel therapeutic targets, immune mechanisms, and long-term consequences.

We seek papers on, but not limited to, the following topics: -Viral infection, especially adenovirus. -Pathogenesis. -Clinical features. -Microbial and host interactions. -Diagnostic markers. -Therapeutic targets. -Immune mechanisms. -Long -term consequences. The result of this call is a relatively comprehensive Research Topic of 5 articles addressing these key aspects.

## Viral infection, especially adenovirus

Li et al. comprehensively reviewed current antiviral drugs targeting human adenoviruses (HAdVs). HAdVs are major pathogens causing respiratory infections. Currently, no effective and safe antiviral therapies have been approved for the clinical treatment of HAdV infections, even among those drugs that have completed preclinical/clinical trials. Nevertheless, numerous compounds and molecules exhibiting anti-HAdV activity have been explored, with several drug candidates currently in clinical development. This paper systematically reviews reports on the *in vitro* and *in vivo* efficacy of these antiviral compounds, evaluates their therapeutic potential, and provides a comprehensive overview of the current landscape in anti-HAdV drug discovery.

## Pathogenesis

The research report by Welham et al. investigated the dysregulated miR-155/BACH1/NRF2 signaling axis in airway epithelial cells of children with Down Syndrome (DS) during RSV infection, and its impact on the expression of hypoxia-responsive genes. This study assessed NRF2-induced antioxidant stress and hypoxia protection genes, particularly heme oxygenase-1 (HO-1), through baseline and post-RSV infection analysis of airway epithelial cells from DS and non-DS children. To investigate DS-specific defects, they focused on miR-155 and BACH1—both encoded on chromosome 21 and jointly regulating the NRF2 signaling pathway and HO-1 expression. RNA sequencing analysis examined the genome-wide hypoxic gene response in control and TS21 AECs at baseline and post-RSV infection. Results revealed that miR-155 enhances NRF2-driven HO-1 expression in euploid AECs by suppressing BACH1. Conversely, TS21 AECs from DS patients exhibited impaired HO-1 induction following miR-155 treatment. This defect was attributed to reduced transcription of the HMOX1 gene encoding HO-1, along with an overall downregulation of hypoxia-responsive genes in TS21 AECs both at the DS baseline state and following RSV infection. This study concludes that severe RSV infection in children with DS may be associated with an inherent defect in their AEC response to hypoxia, including NRF2-driven cell-protective enzymes such as HO-1. These findings provide new insights into the pathophysiological mechanisms of RSV and point toward potential therapeutic targets for children with DS.

## Clinical features

The research article by Stein et al. primarily examined exercise capacity (EC) in pediatric and adolescent patients with post-COVID syndrome (PCS) and its relationship with quality of life. This prospective, single-center, cross-sectional study analyzed exercise capacity, quality of life, and clinical parameters in 29 pediatric patients. Results showed that primary sequelae included reduced subjective exercise capacity (92.4%), shortness of breath (64.2%), inattention (60.4%), and wheezing (47.2%). Overall quality of life (Kindl-R total score) was 89.2% ± 17.3% of normal, self-rated physical health was 60.7% ± 30.4% of normal, and emotional health was 85.1% ± 23.2% of normal. PCS patients were categorized into functional decline and functional recovery groups based on cardiopulmonary recovery. No significant differences existed between groups in age, weight, height, muscle mass, fat percentage, BMI, pulmonary function, neuropsychiatric symptoms, or health status. Although maximum workload and peak oxygen uptake showed significant group differences, lactate levels and self-reported exercise intensity were comparable. However, patients in the functional decline group exhibited significantly shorter recovery times from SARS-CoV-2 infection compared to the functional training group. Pediatric post-COVID syndrome correlates with diminished cardiopulmonary function, which is significantly influenced by post-infection time but does not appear to affect patients' quality of life or self-esteem.

A prospective cohort study conducted in Suqian City, Jiangsu Province, China, from 2023 to 2024 by Li et al. aimed to investigate the pathogen spectrum, epidemiological characteristics, and associated risk factors of acute respiratory infections (ARIs) in infants and young children. The researchers aimed to investigate the frequency and types of respiratory pathogens associated with acute respiratory diseases in infants and young children during this period. A cohort of healthy infants aged 2 months was established in three counties of Suqian City, Jiangsu Province, China. Results showed that 804 infants were invited to participate between February 7 and April 17, 2023. Among them, 796 participants completed enrollment, with a median age of 71.0 days. Common respiratory pathogens detected at baseline included *Staphylococcus aureus* (17.9%), cytomegalovirus (CMV) (16.0%), and *Acinetobacter baumannii* (15.3%). The most prevalent pathogens were CMV (43.7%), *Acinetobacter baumannii* (35.6%), and human rhinovirus (HRV) (29.3%). Among 50 hospitalized ARI cases, HAdV and human RSV (18 cases, 36.0%) were the most frequently detected pathogens. These data contribute to a deeper understanding of the epidemiological characteristics of respiratory pathogens in infants and young children during 2023–2024. Among non-hospitalized acute respiratory infection cases, cytomegalovirus, *Acinetobacter baumannii*, and high-risk respiratory viruses showed the highest detection rates. In contrast, adenovirus and RSV were more frequently detected in hospitalized acute respiratory infection cases. These findings indicate that shifts in pathogen spectrum are closely associated with disease severity, underscoring the necessity of developing targeted prevention and control strategies.

The case report published by Wei et al. primarily examined the clinical manifestations, imaging characteristics, treatment response, and prognosis of viral infections exacerbating pulmonary injury and fibrosis in pediatric patients with diffuse alveolar hemorrhage (DAH). This report describes three cases of viral infection-induced worsening of pulmonary injury and fibrosis in children with diffuse alveolar hemorrhage. To our knowledge, this is among the first literature to document viral infection-induced pulmonary injury and fibrosis in children.

## Concluding remarks

Given the significant impact of viral infections in children, we are grateful to all contributors and reviewers for their vital support in assembling this timely Research Topic and hope readers find valuable insights within these articles.
